# Curcumin derivative C212 inhibits Hsp90 and eliminates both growing and quiescent leukemia cells in deep dormancy

**DOI:** 10.1186/s12964-020-00652-4

**Published:** 2020-09-29

**Authors:** Bi Liu, Yunzhu Shen, Huafang Huang, Kimiko Della Croce, Min Wu, Yingjuan Fan, Yang Liu, Jianhua Xu, Guang Yao

**Affiliations:** 1 School of Pharmacy, Fujian Provincial Key Laboratory of Natural Medicine Pharmacology, Fujian Medical University, Fuzhou, 350122 China; 2 Department of Molecular and Cellular Biology, University of Arizona, Tucson, AZ 85721 USA; 3 The Second Affiliated Hospital of Fujian Medical University, Quanzhou, 362000 Fujian China; 4 Arizona Cancer Center, University of Arizona, Tucson, AZ 85719 USA

**Keywords:** Leukemia, Relapse, Quiescence, Dormancy, Curcumin derivative, Hsp90, Apoptosis, Protein aggregation

## Abstract

**Background:**

Relapsed leukemia following initial therapeutic response and remission is difficult to treat and causes high patient mortality. Leukemia relapse is due to residual quiescent leukemia cells that escape conventional therapies and later reemerge. Eliminating not only growing but quiescent leukemia cells is critical to effectively treating leukemia and preventing its recurrence. Such dual targeting therapeutic agents, however, are lacking in the clinic. To start tackling this problem, encouraged by the promising anticancer effects of a set of curcumin derivatives in our earlier studies, we examined in this work the effects of a 4-arylmethyl curcumin derivative (C212) in eliminating both growing and quiescent leukemia cells.

**Methods:**

We analyzed the effects of C212 on the growth and viability of growing and quiescent leukemia cells using MTS, apoptosis, cell cycle and cell tracking assays. The effects of C212 on the quiescence depth of leukemia cells were measured using EdU incorporation assay upon growth stimulation. The mechanisms of C212-induced apoptosis and deep dormancy, particularly associated with its inhibition of Hsp90 activity, were studied using molecular docking, protein aggregation assay, and Western blot of client proteins.

**Results:**

C212, on the one hand, inhibits growing leukemia cells at a higher efficacy than curcumin by inducing apoptosis and G2/M accumulation; it, on the other hand, eliminates quiescent leukemia cells that are resistant to conventional treatments. Furthermore, C212 drives leukemia cells into and kills them at deep quiescence. Lastly, we show that C212 induces apoptosis and drives cells into deep dormancy at least partially by binding to and inhibiting Hsp90, leading to client protein degradation and protein aggregation.

**Conclusion:**

C212 effectively eliminates both growing and quiescent leukemia cells by inhibiting Hsp90. The property of C212 to kill quiescent leukemia cells in deep dormancy avoids the risk associated with awaking therapy-resistant subpopulation of quiescent leukemia cells during treatments, which may lead to the development of novel therapies against leukemia relapse.

Video abstract

## Background

Conventional chemotherapy drugs target fast-growing cells and induce cell death or cell cycle arrest, typically by damaging DNA or microtubules or inhibiting enzymes required for cell proliferation [1–3]. These drugs, e.g., doxorubicin, paclitaxel and topotecan, are ineffective against slow-growing or quiescent cancer cells that do not undergo active DNA replication and cell division [4, 5]. Accordingly, quiescent cancer cells, including but not limited to cancer stem cells, escape conventional chemotherapies; they often reemerge after a period of dormancy, causing cancer relapse and metastasis that are exceedingly difficult to treat and lead to high patient mortality [6–8].

The ability to eliminate both growing and quiescent cancer cells is critical to treating cancer and preventing cancer recurrence [9, 10]. In contrast to a long list of chemotherapy drugs against growing cancer cells, effective agents eliminating quiescent cancer cells are lacking in the clinic [5, 10]. How to eradicate quiescent slow-growing cancer cells has only begun to be explored, primarily from metabolic and epigenetic angles [11–14]. Recently, we identified two natural compounds, ergosterol peroxide and ganodermanondiol, from the medical mushroom *Ganoderma lucidum* [15]. We showed that these two compounds eliminated quiescent slow-cycling cells by pushing cells to shallow quiescence and exposing them to cytotoxic effects [15]. Awaking quiescent cancer cells to kill presents a strategy to prevent cancer recurrence [16]. However, if some of these quiescent cancer cells develop and acquire therapy resistance during dormancy [4, 10], waking them can be risky.

Can we identify therapeutic agents that eliminate both growing and quiescent cancer cells, without the risk of awakening the latter from dormancy during the treatment? In this work, we studied such potential effects of a curcumin (diferuloylmethane) derivative. Curcumin is the primary natural polyphenol found in the rhizome of *Curcuma longa* (turmeric) and others Curcuma spp. [17]; it exhibits pharmacological effects on a wide range of human diseases [17, 18]. Particularly, it inhibits cancer growth in the reproductive, digestive, urinary, pulmonary, nervous, skeletal, skin, lymphatic, and immune systems, attributing to its immunomodulatory, anti-inflammatory, antioxidant, pro-apoptotic, and antiangiogenic properties [19–24]. At the molecular level, curcumin interacts with multiple cellular pathways: it inhibits NF-κB, Akt/PI3K, and MAPK pathways and enhances p53 activity, to name a few [20, 21]. Recent work [25, 26], including ours [27], showed that curcumin suppresses tumor growth by inhibiting the molecular chaperone function of heat shock protein 90 (Hsp90). Hsp90 chaperone stabilizes a large group of client proteins, including those essential for tumor growth and survival (e.g., Her2, BCR-ABL, and Akt) [28–30]. Accordingly, small molecular drugs that inhibit Hsp90, causing the degradation of Hsp90 client proteins, have exhibited anticancer effects [31–33]. Inhibiting Hsp90 also increases protein aggregation that in turn induces deep quiescence in both bacteria and neural stem cells [34, 35].

Following the anticancer effect of curcumin, we [36–40] and others [41–43] have designed and synthesized curcumin derivatives to address the low bioavailability of curcumin and further improve its anticancer efficacy. Some of these curcumin derivatives (e.g., C086 and C1206) in our earlier studies preserved the Hsp90 inhibition function of curcumin and have shown promising effects against chronic myeloid leukemia (CML) cells [37, 38] and colon cancer cells and xenograft tumors [36]. Here we report that a novel curcumin derivative, C212, exhibits a dual function in eliminating both growing and quiescent leukemia cells; it eliminates quiescent leukemia cells in deep dormancy without waking them up, presenting an attractive approach to prevent leukemia recurrence.

## Materials and methods

### Reagents

C212 was synthesized in our laboratory as described previously [39]. Paclitaxel was purchased from LC Laboratories (P-9600), Topotecan from Sigma (T2705), Doxorubicin from Cayman (15007), and 17-AAG from APExBIO (A405410). The cloning, expression, and purification of the histidine (His)-targeted yeast full-length Hsp90 (1–732, 90 kDa), N-terminus of Hsp90 (N-Hsp90, 1–236, 25 kDa), middle region of Hsp90 (M-Hsp90, 272–617, 40 kDa), and C-terminus of Hsp90 (C-Hsp90,629–732, 15 kDa) were performed as described in previous work [44].

### Cell culture and quiescence induction

K562, HL60, SW620, and MCF-7 cells were cultured in RPMI-1640 medium (Corning, 10040CV) containing 10% bovine growth serum (BGS; Hyclone, SH30541.03). HCT116 cells were cultured in McCoy’s 5A medium (Corning, 1005CV) containing 10% BGS. HT-29, SGC7901, and HepG2 cells were cultured in Dulbecco’s Modified Eagle Medium (DMEM; Hyclone, SH30022.01) containing 10% BGS. To induce quiescent or slow-growing leukemia cells, normal growing cells were spun down, washed once, and plated (in 12-well plates) in the starvation medium: HL60, serum-free DMEM (Corning, 15–013-CV, without glutamine), for 12 h; K562, serum- and amino acid-free Earle’s balanced salt solution EBSS (Gibco, 24,010,043), for 36 h. To induce quiescence exit and cell cycle re-entry, starved leukemia cells were switched to serum stimulation medium: HL60, DMEM (with glutamine) containing 2.5% BGS; K562, EBSS containing 2.5% BGS. To induce quiescent or slow-growing colon cancer cells, normal growing HCT116 and SW620 cells were seeded in 12-well plates and incubated overnight in culture media (see above), then starved in serum- and amino acid-free EBSS for 12 and 24 h, respectively.

### Cell growth/viability MTS assay

**Fig. 1:**
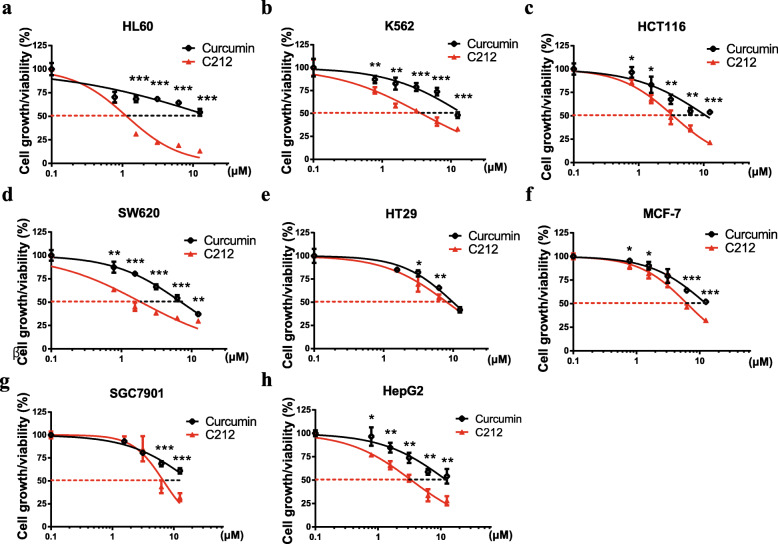
C212 inhibits the growth of a variety of cancer cells. **a**-**h** Growing cancer cells seeded in 96-well plates were treated with curcumin and C212, respectively, at the indicated doses for 48 h. Cell growth was measured with MTS assay in leukemia cell lines HL60 and K562 (**a** and **b**), colon cancer cell lines HCT116, SW620, and HT29 (**c**, **d**, and **e**), breast cancer cell line MCF-7 (**f**), gastric cancer cell line SGC7901 (**g**), and liver cancer cell line HepG2 (**h**). Error bar, SEM (*n* = 3); * *p* < 0.05, ** *p* < 0.01, *** *p* < 0.001 (C212 vs. curcumin; see Methods for statistical analysis detail; the same below)

Cells were seeded in 96-well plates and cultured in 100 μl medium with C212 or other drugs at the indicated doses and durations in figure legend (Figs.1, 4, and S4–5); 20 μl CellTiter stock solution (Promega, G3510) was added into each well, followed by a 3-h incubation at 37 °C. The absorbance of each well was measured at 490 nm, with the absorbance of wells containing medium and CellTiter only set as the background control (A_background_) and the absorbance of wells containing cells treated with vehicle set as the vehicle control (A_control_). Cell growth/viability = (A_treatment_ - A_background_)/(A_control_ - A_background_)*100%.

### Apoptosis assays

The annexin V-FITC/PI staining assay was carried out according to the manufacturer’s protocol (BD, 556547). Briefly, leukemia cells were spun down, washed once in DPBS, resuspended in 100 μL Annexin-V/PI staining buffer, and kept in the dark for 15 min at 37 °C; cells were then washed once with 200 μl wash buffer provided by the kit, followed by FITC-PI fluorescence intensity measurement using flow cytometry. To assess mitochondrial membrane potential (MMP) depolarization during early apoptosis, leukemia cells were spun down, resuspended in 300 μL JC-1 staining solution (KeyGEN Biotech, KGA603), and kept in the dark at room temperature for 10 min; cells were washed twice with the wash buffer provided by the kit, followed by JC-1 fluorescence intensity measurement using flow cytometry. JC-1 is a cationic dye that accumulates in mitochondria and forms red fluorescent aggregates at high MMP; a decrease of JC-1 red fluorescence indicates MMP depolarization.

### Cell cycle and EdU incorporation assays

To assess DNA content, leukemia cells were spun down and resuspended in 500 μl 70% ethanol for overnight at − 20 °C; cells were then washed once with 300 μl cold DPBS, resuspended in 300 μl DNA-staining solution containing propidium iodide (PI; Sigma, P4170) and RNase A (Sigma, R6513) at final concentrations of 50 μg/ml in DPBS, incubated in the dark for 30 min at 37 °C, followed by PI intensity measurement using flow cytometry. To assess EdU incorporation during DNA synthesis after cell cycle re-entry from quiescence, 2 μM EdU was included in serum stimulation medium for 43 and 24 h, respectively, for HL60 and K562 cells, followed by click-iT EdU assay according to the manufacturer’s protocol (Life Technologies, C10418) [45]; EdU intensity was measured using flow cytometry.

### Live cell tracking staining

To track cell division status, cells were stained with a cell tracking dye CFSE (Invitrogen, C1157) according to the manufacturer’s protocol. Briefly, growing leukemia cells were washed once with DPBS and incubated with 500 μl 1x CFSE in DPBS at 37 °C for 20 min. Cells were then washed once with culture medium, and returned to previous culture condition before the staining. Cells were harvested at indicated time points (Fig. 3a), and CFSE dye intensity was measured using flow cytometry.

### Western blot

Whole-cell lysates were prepared with RIPA lysis buffer as described previously [37]. Approximately 40 mg of protein lysate per sample was loaded and separated in 8–12% SDS-PAGE gel and then transferred to PVDF membrane (Roche, 03010040001); membrane was blocked with 5% non-fat dry milk for 1 h and then incubated with primary antibodies at 4 °C overnight, followed by secondary antibody incubation for 2 h at room temperature and imaging. Primary antibody were purchased from Cell Signaling Technology (CST): Caspase 9 (9508S), Caspase 7 (9492S), Caspase 3 (9662S), PARP (9542 T), Cleaved-Caspase 9 (7237S), Cleaved-Caspase7 (8438S), Cleaved-Caspase 3 (9664S), Cleaved-PARP (5625S), Cytochrome C (Cyt,c, 4280S), Akt (4691S), p-AKT (4060S), Raf 9422S), p-Raf (9421S), Mek (4694S), p-Mek (2338S), Erk (4695S), p-Erk (4377S), Cyclin B1 (4135S), Cdc2 (9116S), p-Cdc2 (4539S), β-actin (4970S), β-actin (MA1–140); secondary antibody goat anti-rabbit IgG-HRP was purchased from Santa Cruz Biotechnology (sc-7074). Western blot bands were quantified using ImageJ with background noise removed using Rolling-Ball correction.

### Hsp90-interaction analysis

For the molecular docking analysis, chain A of the crystal structure of an Hsp90-Sba1 closed chaperone complex was extracted from Protein Data Bank (PDB, ID: 2CG9), from which structures of Hsp90 C- and N-terminus were derived [46]; the bindings between Hsp90 domains and curcumin/C212 were simulated using the Surflex-Dock program (SYBYL-X v1.3). For the quenching assay of intrinsic Hsp90 fluorescence, a C212 solution (0 to 50 μM in 0.2% DMSO) was successively added into 2.0 mL Hsp90 solution (5.0 μM in PBS, pH 7.6); fluorescence intensity was recorded from 290 to 500 nm at 303 Kelvin using a Cary Eclipse spectrofluorometer (Varian/Agilent).

### Protein aggregation assay

The accumulation of protein aggregates in the cell was assessed using the ProteoStat Aggresome detection kit (Enzo, ENZ-51035-K100) according to the manufacturer’s protocol. Briefly, leukemia cells were spun down, fixed with 200 μl 4% paraformaldehyde at room temperature for 20 min, and permeabilized with 200 μl assay buffer containing 0.5% Triton-X100 and 3 mM EDTA in PBS on ice for 20 min. ProteoStat staining solution was added to cell suspension at 1:5000 and incubated at room temperature for 30 min, followed by ProteoStat fluorescence measurement using flow cytometry.

### Data and statistical analysis

Cell growth/viability data were analyzed using Prism 6.0 (GraphPad), including curve fitting and IC50 calculation (using a non-linear regression fit, log(inhibitor) vs. normalized response – variable slope) as well as AUC calculation (using the trapezoidal rule). Flow cytometry data were analyzed using FlowJo v10.3 (BD). Statistical significance between two independent groups of measurements was determined using unpaired Student’s *t*-test; 1-tailed tests were performed to assess C212-induced unilateral difference (increase or decrease) over control in all figures, except for Fig. S3–5 where 2-tailed tests were performed to assess the mixed change patterns observed.

## Results

### C212 inhibits the growth of a variety of cancer cells more effectively than curcumin

C212, (1E,6E)-4-(2-Chlorinebenzyl)-1,7-bis(3-pyridine)-1,6-heptadiene-3,5-dione (Fig. S1A), was synthesized as one of several 4-arylmethyl curcumin analogues in our previous study [39]. Here we applied C212 and curcumin to a variety of cancer cell lines, including leukemia (AML HL60 and CML K562), colon cancer (HCT116, SW620, and HT29), breast cancer (MCF-7), gastric cancer (SGC7901), and liver cancer (HepG2). We found that both C212 and curcumin dose-dependently suppressed the growth of these cancer cell lines as shown in MTS assays (Fig. 1a-h). C212 further exhibited a stronger growth-inhibition effect than curcumin in each tested cancer cell line. Compared to curcumin, C212 exhibited (i) a lower IC50, the concentration required to inhibit cell growth by 50% (Fig. 1a-h; Table 1), and (ii) a smaller area under the curve (AUC in Fig. 1a-h; quantified in Table 1) that indicated a stronger cumulative growth-inhibition effect across the tested dose range.

**Table 1:** IC50 values of C212 and curcumin in cancer cell lines (48-h treatment; Cur, curcumin)

		HL-60	K562	HCT116	SW620	HT29	MCF-7	SGC7901	HepG2
Growth% under C212 vs. Cur (*p*-value)	0.8 μM	1.6E-01	9.7E-03	3.3E-02	9.4E-03		2.8E-02		3.5E-02
	1.6 μM	1.1E-03	7.4E-03	3.5E-02	5.6E-04	4.8E-01	4.7E-02	1.7E-01	4.9E-03
	3.2 μM	2.3E-07	2.7E-03	1.2E-02	3.3E-04	6.0E-02	2.2E-01	3.2E-01	3.2E-03
	6.3 μM	7.0E-05	4.3E-04	1.5E-03	8.1E-04	4.6E-03	4.0E-04	1.3E-03	5.4E-03
	12.5 μM	1.2E-03	7.1E-03	3.6E-05	9.5E-03	4.6E-01	1.3E-04	7.3E-04	6.2E-03
IC 50 (μM)	C212	1.2	3.7	3.4	1.9	8.2	6.3	6.7	3.4
	Cur	17.6	14.8	11.0	7.1	10.0	12.6	18.9	12.3
	C212/Cur	0.07	0.25	0.31	0.27	0.82	0.50	0.36	0.27
Mean (IC50 C212/Cur)		0.16		0.47			0.50	0.36	0.27
AUC	C212	108.1	140.4	145.2	122.1	173.5	164.0	174.7	140.5
	Cur	155.5	174.6	173.2	162.9	180.0	179.7	183.4	175.9
	C212/Cur	0.70	0.80	0.84	0.75	0.96	0.91	0.95	0.80
Mean (AUC C212/Cur)		0.75		0.85			0.91	0.95	0.80

Among the tested cell lines, leukemia cells as a group were the most sensitive to C212. In leukemia cells (HL60 and K562), the mean IC50 ratio (C212 vs. curcumin) was 0.16 as compared to 0.27–0.50 in other cancer cell types (Table 1), and the mean AUC ratio (C212 vs. curcumin) was 0.75 as compared to 0.80–0.95 in other cancer cell types (Table 1). These results indicated that leukemia cells underwent stronger growth-inhibition effects than other cancer cells did under C212 (compared to curcumin). We therefore focused on leukemia cells in subsequent C212 study.

### C212 inhibits leukemia cell growth by inducing apoptosis and G2/M accumulation

**Fig. 2:**
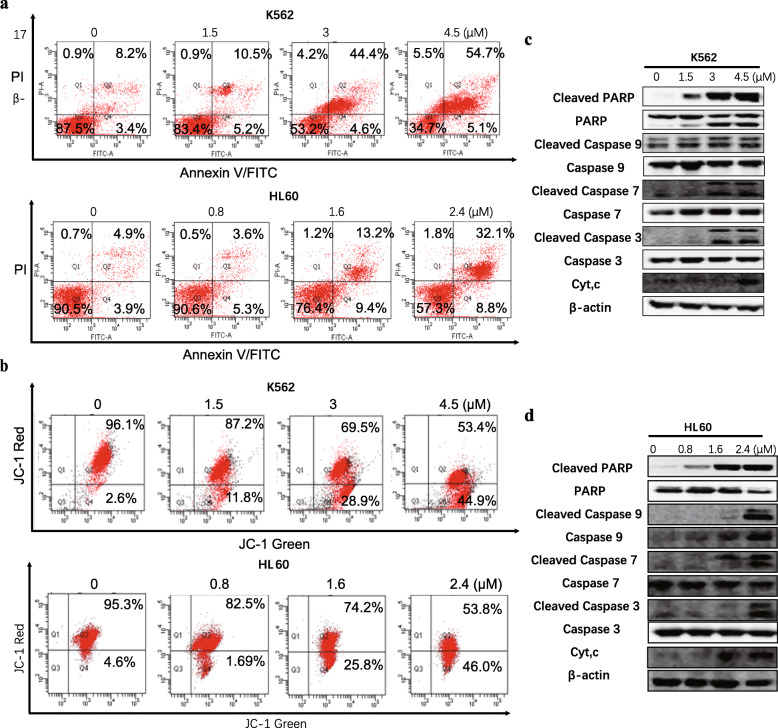
C212 induces leukemia cell apoptosis. Growing leukemia cells K562 and HL60 were treated with C212 at the indicated doses for 24 h and subjected to apoptosis assay with Annexin-V & PI staining (**a**), mitochondrial membrane potential MMP assay with JC-1 staining (**b**), and Western blot of apoptosis marker proteins (in K562, **c**; in HL60, **d**)

We next tested the inhibitory effect of C212 on leukemia cell growth. It appeared to be twofold. First, C212 dose-dependently induced apoptosis in HL60 and K562 cells. With an increasing C212 concentration, 1) the subpopulation of cells exhibiting the characteristic externalization of inner membrane phospholipids (Annexin V-positive) and membrane compromise (PI-positive) increased monotonically in K562 (Fig. 2a, top) and HL60 cells (Fig. 2a, bottom); 2) the subpopulation of cells exhibiting the characteristic depolarized mitochondrial membrane potential (with reduced red fluorescence of JC-1 staining) increased monotonically in K562 (Fig. 2b, top) and HL60 cells (Fig. 2b, bottom); 3) the amounts of cleaved forms of PARP, caspase-9, caspase-7 and caspase-3, as well as the amount of cytochrome C (Cyt-C) in cell lysates that reflected primarily the level of released Cyt-C from mitochondria into cytoplasm, increased with C212 in K562 (Fig. 2c) and HL60 cells (Fig. 2d).

**Fig. 3:**
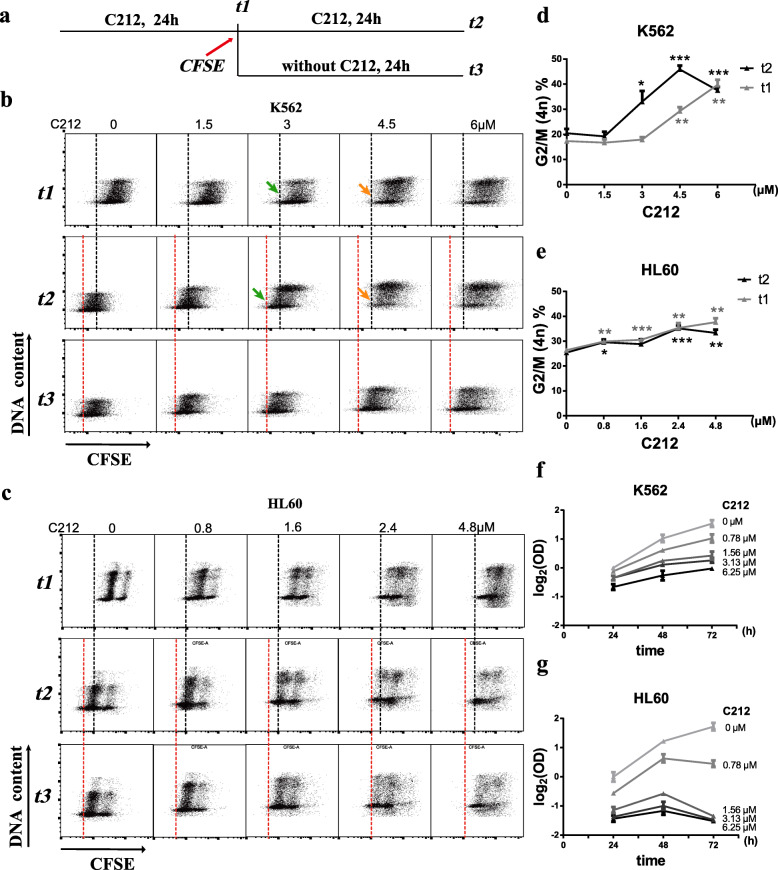
C212 induces leukemia cell G2/M accumulation. **a** Experiment scheme to identify G2/M-arrested cells. Cells were stained with the cell tracing dye CFSE at *t1* (following a 24-h C212 treatment), subsequently harvested at *t1, t2* (following another 24-h C212 treatment)*,* or *t3* (24 h after *t1* without C212 treatment), and subjected to PI DNA staining. **b** and **c** Double staining with CFSE (x-axis) and PI (y-axis) at *t1, t2,* or *t3* in K562 (**b**) and HL60 cells (**c**). Black dash lines mark the left boundaries of cell populations at *t1*; red dash lines mark the half CFSE values of the corresponding black dash lines. **d** and **e** G2/M percentages at *t2* versus *t1* in K562 (**d**) and HL60 cells (**e**), quantified from the PI staining in **b** and **c**, respectively, based on DNA/cell cycle analysis in FlowJo. Error Bar, SEM (n = 3); * *p* < 0.05, ** *p* < 0.01, *** *p* < 0.001 (over control at 0 μM). **f** and **g** Cell growth/viability of K562 (**f**) and HL60 cells (**g**) indicated by the Optical density (OD) values in MTS assay (y-axis, normalized to the 0 μM control) under C212 treatments at indicated doses over time (24–72 h, x-axis)

Second, C212 dose-dependently increased the proportion of cells accumulated at G2/M phase in both K562 and HL60 cells (Fig. S1 B and C). This result suggested in cells that survived apoptosis (Fig. 2), (a) G2/M progression was slowed down or arrested, which was consistent with the observed downregulations of Cdc2 and Cyclin B1 (G2/M “triggers”) under C212 treatment (Fig. S1D), and/or (b) cells were more resistant to C212 cytotoxicity in G2/M than in other cell cycle phases. To test these potential mechanisms and examine how G2/M-accumulated cells behave under C212 over time, we first needed to identify and track C212-induced G2/M-arrested cells, if any. To this end, we labeled cells with a cell trace dye CFSE after C212 treatment for 24 h (*t1* point, Fig. 3a), we then followed cells under C212 for another 24 h (*t2* point, Fig. 3a). At *t1* and *t2*, a subset of cells was harvested, followed by propidium iodide (PI) DNA staining. Cells that were G2/M-arrested at *t1* would not divide and thus retain a 4n DNA content and the same CFSE intensity (*I*) at *t2*, *I*_*t2*_ *= I*_*t1*_. In contrast, divided cells after *t1* had the CFSE dye split into two daughter cells, each with *I*_*t2*_ = ½*I_*t1.*_

In K562 cells, when treated with C212 at the lower dose range (0–3 μM), most cells were not arrested but divided: at *t1*, cells were to the right of the black dash line (*I*_*t1*_); at *t2*, they shifted to the right of the red dash line (*I*_*t2*_), with *I*_*t2*_ = ½*I_*t1*_ (green arrows pointed, an example at 3 μM; Fig. 3b). In contrast, with C212 at the higher dose range (4.5 and 6 μM), most K562 cells were arrested after *t1* and remained to the right of the black dash line at *t2* (orange arrow pointed, an example at 4.5 μM; Fig. 3b). Furthermore, the G2/M% in K562 cells increased significantly at high C212 doses over control (at both *t1* and *t2*, Fig. 3d) and with time (*t2* vs. *t1*, Fig. 3d), indicating a C212-induced G2/M arrest. In comparison, most HL60 cells did not arrest their cell cycle, but divided at tested C212 doses and shifted to the right of red dash line by *t2* (Fig. 3c). Consistently, the G2/M% in HL60 cells increased relatively modestly with increasing C212 doses (at both *t1* and *t2*, Fig. 3e) and did not increase over time (*t2* vs. *t1*, Fig. 3e), suggesting a partial slowdown but not arrest at G2/M in most HL60 cells.

Were G2/M-arrested cells more resistant to C212 cytotoxicity than non-arrested ones? The significant enrichment of C212-induced G2/M-arrested cells in K562 but not HL60 cells, correlated with the over 3-fold larger C212 IC50 in K562 than in HL60 (Table 1), was supportive of this notion. To test it further, we did a cell growth/viability time-course analysis of K562 and HL60 cells under C212 treatment. In HL60 cells, the relative cell growth (in MTS assay) under C212 (0.78–6.25 μM) between 24 and 48 h were lower than the vehicle control but still positive (Fig. 3g), while that between 48 and 72 h became negative (Fig. 3g), indicative of cell death surpassing growth. In comparison, the relative cell growth of K562 cells under C212 treatment remained positive (24 to 48 to 72 h; Fig. 3f). This discrepancy between K562 and HL60 cells further supported the notion that G2/M-arrested cells, enriched over time in K562 but not HL60 cells, were more resistant to C212 killing than non-arrested ones; they largely survived C212 treatment at least for 72 h (while cell death surpassed growth in HL60 cells after 48 h). Lastly, we note that the C212-induced G2/M-arrested cells were reversible – as seen in Fig. 3b, when C212 was removed, most cells arrested at *t1* divided and shifted from the right of the black dash line to the right of the red dash line after 24 h (*t3*, Fig. 3b).

### C212 eliminates quiescent leukemia cells in contrast to chemotherapy drugs

**Fig. 4:**
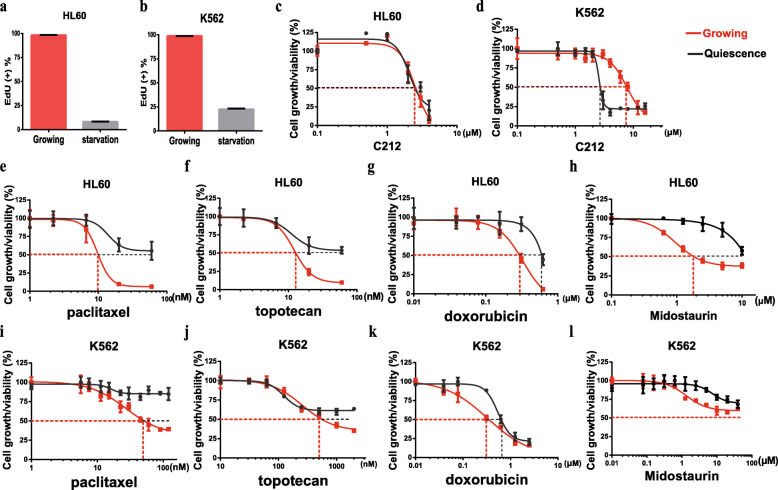
C212, in contrast to chemotherapy drugs, effectively eliminates dormant leukemia cells. **a**, **b** Serum starvation-induced quiescence and slow-growing state. HL60 (**a**) and K562 (**b**) cells were deprived of serum for 12 and 36 h, respectively, to induce quiescence/slow-growth; EdU (2 μM) was added to the medium and cells were further incubated for 43 h (**a**) and 24 h (**b**), followed by a Click-iT assay to assess EdU incorporation in cells. Growing: control cells not deprived of serum but otherwise under the same treatment. Error bar, SEM (*n* = 2). **c**-**l** Growing and serum starvation-induced (as in **a** and **b**) quiescent cells (HL60, **c**, **e**-**h**; K562, **d**, **i**-**l**) were treated with C212, paclitaxel, topotecan, doxorubicin, and midostaurin at the indicated doses for 48 h and subjected to MTS assay. Error bar, SEM (*n* = 3)

Different from growing cells, quiescent cells are resistant to conventional chemotherapies that target cell proliferation machinery involving DNA replication and cell division. To test the cytotoxicity of C212 on quiescent leukemia cells, we first induced HL60 and K562 cells to quiescence or slow-growth by serum starvation: a 12-h serum starvation reduced the percentage of actively proliferating (EdU + %) HL60 cells from 98.3% in growing condition to 8.1% (Fig. 4a); for K562 cells, which appeared to be less sensitive to serum growth signals than HL60, a 36-h serum starvation reduced EdU + % from 98.8% in growing condition to 22.5% (Fig. 4b). As expected, chemotherapy drugs were inefficient in killing quiescent and slow-growing leukemia cells: the IC50 values of paclitaxel, topotecan, and doxorubicin in quiescent cells were several folds higher than the IC50 values in growing cells (HL60, Fig. 4e-g and Table 2; K562, Fig. 4i-k and Table 2). Similarly, Midostaurin [47], a targeted therapy drug against AML, was also inefficient in killing quiescent but growing leukemia cells, with the dose required to kill the same percentage of quiescent cells several fold higher than that of growing cells in both AML HL60 (Fig. 4h) and CML K562 (Fig. 4l) cells. By contrast, C212 exhibited the same IC50 (2.3 μM) in both quiescent and growing HL60 cells (Fig. 4c). Furthermore, in K562 cells, the IC50 of C212 in quiescent cells (2.8 μM) was only 36% of that in growing ones (7.7 μM), indicating noticeably stronger cytotoxicity in quiescent than growing condition (Fig. 4d). Consistently, C212 exhibited a smaller AUC ratio (quiescence vs. growing) in both HL60 and K562 cells compared to paclitaxel, topotecan, doxorubicin, and midostaurin (Fig. 4c-d vs. e-l; Table 2), indicating stronger cumulative cytotoxicity of C212 against quiescent leukemia cells. We note our data also suggested that quiescent K562 cells (induced by serum starvation) differ from G2/M-arrested counterparts (induced at high C212 doses, Fig. 3b) – at these C212 doses (4.5 or 6 μM), the vast majority of quiescent K562 cells died, while most growing cells survived (Fig. 4d), particularly the G2/M-arrested ones (Fig. 3d). Put together, we concluded that in contrast to conventional chemotherapy drugs, C212 preferentially kills quiescent leukemia cells.

**Table 2:** IC50 values of chemotherapy and targeted therapy drugs vs. C212 in quiescent (Q) and growing (G) leukemia cells (48-h treatment)

Drugs	HL60						K562					
	IC50			AUC			IC50			AUC		
	Q	G	Q/G	Q	G	Q/G	Q	G	Q/G	Q	G	Q/G
Paclitaxel/nM	62.2	10.8	5.76	148.2	103.8	1.43	> 120	58.5	> 2.05	191.8	162.7	1.18
Topotecan/nM	57.8	13.3	4.35	142.8	112.9	1.27	> 2000	752.9	> 2.66	182.9	173.4	1.06
Doxorubicin/μM	0.6	0.3	2.00	162.5	139.3	1.17	0.7	0.4	1.75	182.0	149.5	1.22
Midostaurin/μM	12.1	2.9	4.17	185.3	133.2	1.39				324.5	300.4	1.08
C212/μM	2.3	2.3	1.00	128.7	124.3	1.04	2.8	7.7	0.36	154.3	178.3	0.87

### C212 drives quiescent leukemia cells into deep dormancy

**Fig. 5:**
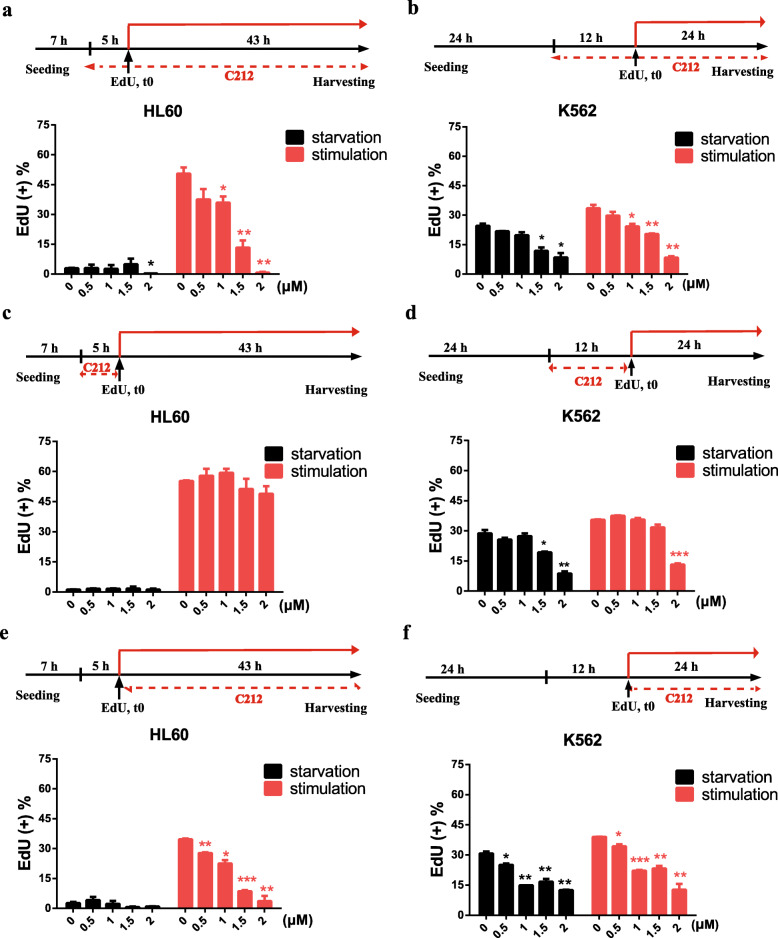
C212 drives leukemia cells into deep quiescence. (**a** and **b**) Leukemia cells were serum-starved for the indicated durations (before t0) to induce quiescence/slow-growth as in Fig. 4 a and b; cells were then either kept in starvation condition (indicated by a black line), or stimulated with 2.5% serum (indicated by a red line) for 43 h in HL60 cells (**a**) or 24 h in K562 cells (**b**), followed by cell harvesting and Click-iT EdU assay. C212 at indicated doses was included in the last segment of starvation and though serum stimulation, as indicated with a red dash line. EdU (2 μM) was added between t0 and harvesting. **c** and **d** Same as **a** and **b**, except that C212 was included only in the last segment of starvation (as indicated with a red dash line). **e** and **f** Same as **a** and **b**, except that C212 was included only during serum stimulation (as indicated with a red dash line). Error bar, SEM (n = 2); * *p* < 0.05, ** *p* < 0.01, *** *p* < 0.001 (over control at 0 μM)

It has been previously reported that arsenic trioxide (As2O3) and certain cytokines (IFN-α, G-CSF) were able to sensitize quiescent leukemia cells to chemotherapy cytotoxicity by reducing quiescence depth and waking them up [9]. Correspondingly, we examined whether C212 altered cell quiescence depth. To measure quiescence depth, we first induced leukemia cells into quiescent or slow-growth by serum starvation (for 12 h in HL60 and for 36 h in K562, same as in Fig. 4a and b); we then stimulated cells with serum (2.5%) and measured the percentages of cells that exited quiescence and re-entered the cell cycle (EdU+): deeper quiescent cells would be harder to exit quiescence and thus have smaller EdU + % than shallower cells. When C212 was included in the last segment of serum starvation and through the serum stimulation phase (indicated with a red dash line, Fig. 5a and b), the EdU + % upon serum stimulation decreased in a C212 dose-dependent manner in HL60 cells (Fig. 5a), from 50.5% (0 μM) to 13.3% (1.5 μM, sublethal according to Fig. 4c); in K562 cells that are less sensitive to serum signals than HL60, the relatively high EdU + % background in serum starvation condition decreased in a C212 dose-dependent manner, as well as the EdU + % upon serum stimulation (Fig. 5b) which decreased from 33.5% (0 μM) to 8.3% (2 μM, sublethal according to Fig. 4d). These results indicated that C212 treatment at increasing sublethal doses drove leukemia cells into deeper quiescence, so that a smaller percentage of quiescent cells was able to exit quiescence and re-enter the cell cycle given the same serum-stimulation condition (2.5%).

We next examined whether the effect of C212 to drive leukemia cells into deeper quiescence was exerted before or during quiescence exit or both. Our previous studies showed that quiescence depth is determined by various cellular activities during serum starvation and stimulation (i.e., before and during quiescence exit, respectively) that affect the serum threshold to activate an Rb-E2F bistable gene switch [45, 48]. When C212 was included in the last segment of serum starvation but not the serum stimulation phase, cell cycle re-entry was not affected in HL60 cells (EdU + % upon serum stimulation showed no statistically significant difference with or without C212; Fig. 5c) and was also not affected in K562 cells until the high C212 dose (2 μM, Fig. 5d). When C212 was included in the serum stimulation phase but not in the last segment of serum starvation, cell cycle re-entry was affected significantly in both HL60 and K562 cells, and EdU + % upon serum stimulation decreased in a C212 dose-dependent manner (Fig. 5e and f). Put together, our data suggested that C212 drove leukemia cells into deep quiescence, and it exerted this quiescence-deepening effect primarily by increasing the serum threshold during quiescence exit.

### C212 inhibits Hsp90 and induces client degradation and protein aggregation in leukemia cells

**Fig. 6:**
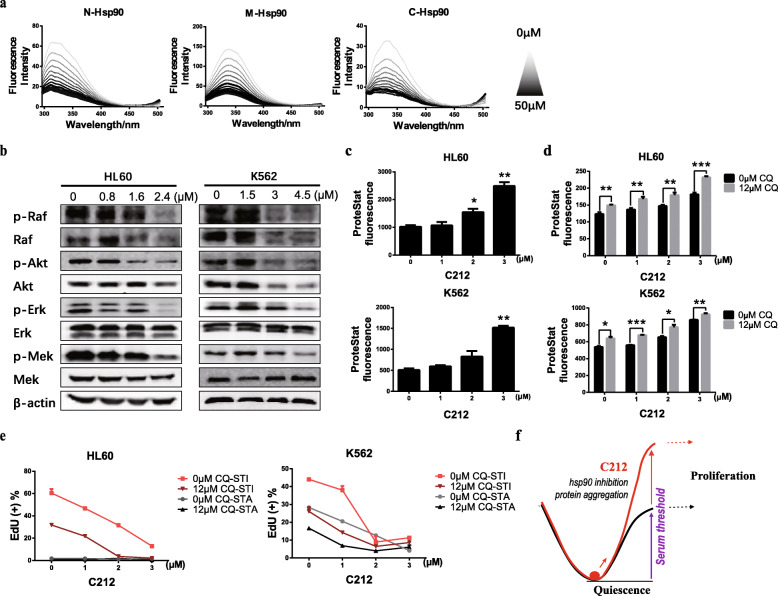
C212 decreases Hsp90 client protein levels and induces protein aggregates. **a** Concentration-dependent quenching effect of C212 (0–50 μM) on the intrinsic fluorescence of N-, M-, and C-domains of Hsp90. Y- and X-axis indicate fluorescence intensity and emission wavelength, respectively. Each curve represents the average of triplicate measurements. **b** Leukemia cells (HL60, left; K562, right) were treated with C212 at the indicated doses for 24 h, followed by Western blot of Hsp90 client proteins in cell lysates. **c** HL60 (top) and K562 (bottom) cells that were serum-starved as in Fig. 4 a and b to induce quiescence/slow-growth were further treated with C212 at the indicated doses for 30 and 36 h, respectively, then subjected to fluorescence-based protein aggregation assay using ProteoStat. Error bar, SEM (n = 2); * *p* < 0.05, ** *p* < 0.01 (over control at 0 μM). **d** Quiescent HL60 (top) and K562 (bottom) cells were treated with C212 at the indicated doses for 30 and 36 h, respectively, in the presence or absence of 12 μM CQ, then subjected to fluorescence-based protein aggregation assay using ProteoStat. Error bar, SEM (n = 2); * *p* < 0.05, ** *p* < 0.01, *** *p* < 0.001. **e** HL60 (left) and K562 (right) cells were serum-starved, treated with C212 at the indicated doses in the presence or absence of CQ, and serum-stimulated as in Fig. 5 a and b, respectively. Cells were then harvested and subjected to Click-iT EdU assay. **f** A proposed model of C212 to deepen quiescence of leukemia cells by inhibiting Hsp90 and inducing protein aggregates, and thus increasing the serum threshold for quiescence exit

As found in our previous work [27], curcumin binds to the α-helix domain of Hsp90 at multiple residues (Fig. S2A) and inhibits the function of Hsp90 in folding and stabilizing client proteins—e.g., kinases that participate in cell proliferation. In our molecular docking analysis, C212 was also able to bind to the C- and N-terminus as well as the middle (M) region of Hsp90 (Fig. S2A). When we carried out fluorescence quenching experiments with purified Hsp90 protein, we found that C212 dose-dependently quenched the intrinsic fluorescence of Hsp90 (excitation/emission = 280/337 nm), which was accompanied with blueshifts of λem curves (Fig. 6a). Specifically, the titration curves of C212 with the N-terminus, M-region, and C-terminus of Hsp90 yielded estimated dissociation constants (K_d_) of 36.3, 36.9, and 15.3, respectively (Table 3), suggesting that C212 interacted with Hsp90.

**Table 3:** Dissociation constant (Kd) values of C212 with different Hsp90 domains

Compound	Fmax	Kd (μM)	R^2^
N-Hsp90	80.8 ± 1.1	36.3 ± 1.0	0.999
M-Hsp90	195.8 ± 2.5	38.7 ± 0.9	0.999
C-Hsp90	32.8 ± 0.9	15.3 ± 1.2	0.995

Through binding to Hsp90, C212 was expected to behave like curcumin and interfere with the Hsp90 function in folding and stabilizing client proteins. Indeed, in both K562 and HL60 cells treated with C212, protein levels of Hsp90 clients Raf, Akt, Erk, and Mek, particularly in their phosphorylated forms, decreased in a C212 dose-dependent manner (Fig. 6b). The C212-induced decreases of Hsp90 client proteins were through proteasome-mediated degradation and could be blocked with a proteasome inhibitor MG-132 (Fig. S2B); they were not caused by decreases in Hsp90 protein level, which did not change or even increased modestly at nearly all tested conditions (Fig. S3 A and B, HL60; Fig. S3 H and I, K562).

We also found that C212 treatment induced the formation of protein aggregates. As seen in both HL60 (Fig. 6c, top) and K562 cells (Fig. 6c, bottom) induced to quiescence by serum starvation, the degree of protein aggregation (as measured by ProteoStat staining) increased in a C212 dose-dependent manner. This result was consistent with the notion that C212 interferes with Hsp90 function, which leads to the accumulation of misfolded proteins. Protein aggregates have been recently shown to drive deep cellular dormancy in both bacteria and neural stem cells [34, 35]. Since lysosomal activities are critical to partially clearing protein aggregates (as seen in quiescent neural stem cells [35]), we expected that blocking lysosomal activities would further increase protein aggregates and deepen quiescence in C212-treated cells. Indeed, co-treating quiescent leukemia cells HL60 and K562 with C212 and a lysosome inhibitor chloroquine (CQ), compared to C212 treatment alone at all tested doses, further increased the degree of protein aggregation (as shown by higher ProteoStat fluorescence intensities, Fig. 6d) and deepened quiescence (as shown by the reduced EdU% both under serum starvation (STA) and stimulation (STI), Fig. 6e).

## Discussion

In this study, we found that C212, a 4-arylmethyl curcumin derivative demonstrates a dual anticancer effect against leukemia cells: it not only inhibits cell growth by inducing apoptosis and G2/M accumulation, but eliminates quiescent cells that are resistant to conventional chemotherapy drugs such as paclitaxel, doxorubicin, and topotecan, as well as the targeted therapy Midostaurin.

Quiescence is a reversible cellular dormancy state that can persist over prolonged periods. Leukemia relapse, despite initial therapeutic responses and remissions, is often due to the reemergence of residual quiescent cells, including leukemia stem cells [37, 49–52]. Therefore, effective therapies targeting and eliminating quiescent leukemic cells are critical to preventing leukemia relapse. In this regard, cytokines such as IFN-α and G-CSF, as well as arsenic trioxide As2O3, have been shown to wake up quiescent leukemia stem cells and sensitize them to chemotherapy [9]. However, some quiescent leukemia cells during long-term dormancy under stressful conditions can undergo genetic or epigenetic changes and further develop resistance to follow-up therapies [4, 10]; awakening these quiescent leukemia cells may therefore cause a risk.

C212 presents a likely safer strategy to prevent leukemia relapse by pushing quiescent leukemia cells into and killing them at deep dormancy, instead of waking them up. The effect of C212 to induce deep quiescence is at least partially due to its binding to Hsp90 and inhibiting Hsp90 function (Fig. 6a and b; Fig. S2A). Hsp90 is an evolutionarily conserved molecular chaperone that participates in stabilizing and activating more than 200 client proteins [30, 53]. Inhibiting Hsp90 function can lead to clients degradation (discussed further below) as well as the aggregation of misfolded proteins. Consistently, C212 dose-dependently induces protein aggregation (Fig. 6c) and drives quiescent leukemia cells into deep dormancy at sublethal doses by creating a higher serum threshold during quiescence exit (Fig. 5), a notion we summarized in Fig. 6f.

Previously, we have found that by inhibiting Hsp90, curcumin and its derivatives induce the degradation of Hsp90 client proteins and correspondingly the growth arrest and apoptosis of leukemia cells [27, 37, 38]. These results were expected as many Hsp90 client proteins, such as kinases and transcription factors, are critical to cellular homeostasis and cell survival against stressful conditions [28–30, 54]. Similarly, C212 binds to and inhibits Hsp90, induces the degradation of Hsp90 client proteins (Fig. 6b), and induces apoptosis in K562 and HL60 leukemia cells (Fig. 2).

A few significant questions are still unanswered and await future research. First, we found different from in leukemia cells, in colon cancer cells C212 eliminated quiescent cells less effectively than growing ones, behaving similar to chemotherapy drugs in this regard (Fig. S4 A-D). This discrepancy again appeared correlated with the ability of C212 to inhibit Hsp90 and induce client protein degradation in quiescence. In quiescent leukemia cells HL60 and K562, we found Hsp90 client protein p-Erk level decreased in a C212 dose-dependent manner either with (Fig. S3 E and L) or without (Fig. S3 D and K) normalizing to Hsp90 level. By contrast, in quiescent colon cancer cells HCT116 and SW620, p-Erk level did not decrease, but increased (although mostly statistically insignificantly) with C212, either with (Fig. S4 I and N) or without (Fig. S4 H and M) normalizing to Hsp90 level. We propose that the ability of C212 to inhibit Hsp90 in quiescence (as in leukemia cells HL60 and K562, but not in colon cancer cells HCT116 and SW620) is a key determinant of whether C212 can effectively eliminate quiescent cells (notion I). On top of this, Hsp70 appeared to also affect the relative sensitivity of quiescent and growing cells to C212. In HL60 and K562 cells (exhibiting C212-induced Hsp90 inhibition and client protein degradation), the increase of Hsp70 level was noticeably higher in growing K562 cells compared to the other three cell populations (quiescent K562 cells as well as growing and quiescent HL60 cells; Fig. S3 J and C), which was consistent with the apparently higher C212-resistance of growing K562 cells than the other three cell populations (Fig. 4c and d) considering the role of Hsp70 in repressing apoptosis [55, 56]. In colon cancer cells HCT116 and SW620 (not exhibiting C212-induced Hsp90 inhibition and client protein degradation, Fig. S4 H-N), Hsp70 level increased with C212 in growing condition but remained undetectable in quiescence (Fig. S4 E and G, HCT116; Fig. S4 J and L, SW620) – still, quiescent colon cancer cells were more resistant to C212 than growing ones (Fig. S4 C and D). These results suggested that when quiescent cells did not exhibit C212-induced Hsp90 inhibition and client degradation, they were more resistant to C212 killing than their growing counterparts, even without the anti-apoptosis protection from Hsp70 as growing cells had (notion II). These two related notions (I and II) need to be studied more extensively and with other cell types in future research, as well as the potential reasons underlying the seemingly cell-type specific effects of C212 (e.g., whether due to differences in intracellular drug availability, suspension vs. adherent environment, or other confounding factors).

Second, we note that inhibiting Hsp90 did not solely account for the action of C212. When we targeted to inhibit Hsp90 using 17-AAG, a classic Hsp90 inhibitor, we found in both HL60 and K562 leukemia cells, growing cells instead of quiescent ones were preferentially eliminated (Fig. S5 A and B). Yet, comparable trends were observed regarding Hsp90 and client protein (p-Erk) level changes in response to 17-AAG and C212 in these cells (Fig. S5 C and J). Particularly, Hsp90 level increased slightly (but mostly statistically insignificantly; Fig. S5 D-F, HL60; Fig. S5, K-M, K562), and p-Erk level decreased significantly (Fig. S5 G-I, HL60; Fig. S5, N-P, K562), with increasing doses of C212, 17-AAG, and their combinations. These results suggested that the action of C212 involved cellular targets beyond Hsp90 alone, a notion consistent with the broad spectrum of molecular targets known to curcumin and its derivatives [20, 21]. For example, C212 may target cell survival factors not associated with Hsp90, or it may directly target some Hsp90 client proteins independent of Hsp90 inhibition (as curcumin does [21, 57]). The exact underlying mechanism(s) await future studies.

## Conclusion

Here, we report that C212, a 4-arylmethyl curcumin derivative, exhibits a dual effect in eliminating both growing and quiescent leukemia cells. C212 inhibits growing leukemia cells at a higher efficacy than curcumin by inducing apoptosis and G2/M accumulation; it also kills quiescent leukemia cells in deep dormancy that are resistant to conventional chemotherapy drugs. The cytotoxicity of C212 against leukemia cells is related to its inhibition of Hsp90, leading to client protein degradation and protein aggregation. We expect that further elucidating the molecular mechanisms underlying the dual effect of C212, particularly its preferential elimination of quiescent leukemia cells, may lead to novel therapeutic strategies in treating leukemia and preventing its recurrence from residual dormant cancer cells.

## Supplementary information

**Additional file 1 : Fig. S1.** C212 induces G2/M cell accumulation. (*A*) Chemical structures of curcumin and C212. (*B* and *C*) Growing leukemia cells were treated with C212 at the indicated doses for 24 h and subjected to cell-cycle analysis with PI staining of DNA content (*B*, K562; *C*, HL60). Error bar, SEM (*n* = 2); * *p* < 0.05, ** *p* < 0.01 (over control at 0 μM). (*D*) Cells were treated with C212 as in *B* and *C* and subjected to Western blot of Cdc2 and cyclin B1 proteins (left, K562; right, HL60). **Fig. S2.** C212 binds to Hsp90 and induces proteasome-dependent client protein degradation. (*A*) Molecular docking simulation of the binding of curcumin, C212, and 17-AAG to Hsp90 at its C-terminus, middle (M-)region, and N-terminus. (*B*) Growing HL60 cells were pretreated with or without C212 (2.4 μM) for 2 h, followed by co-treatment with or without MG132 (1 μM) for 12 h; whole-cell lysate was then subjected to Western blot. **Fig. S3** Growing and serum starvation-induced quiescent HL60 (*A*) and K562 (*H*) cells were treated with C212 at the indicated doses for 24 h, followed by Western bolt assay. Protein levels of Hsp90 (*B*, HL60; *I*, K562), Hsp70 (*C*, HL60; *J*, K562), and Hsp90 client protein p-Erk (*D*, HL60; *K*, K562) were normalized to β-actin loading control and plotted against C212 doses. Also shown are p-Erk levels normalized to corresponding Hsp90 levels at tested C212 doses (*E*, HL60; *L*, K562). Insets in *D-L* show the enlarged bar graphs corresponding to Quiescence. Error bar, SEM (*n* = 2); * *p* < 0.05, ** *p* < 0.01, *** *p* < 0.001 (over control at 0 μM). **Fig. S4** HCT116 (*A*) and SW620 (*B*) cells were serum-starved for 12 and 24 h with EBSS, respectively, to induce quiescence/slow-growth; EdU (10 μM) was added to the medium, and cells were further incubated for 24 h, followed by EdU incorporation assay. Growing: control cells not deprived of serum but otherwise under the same treatment. Error bar, SEM (n = 2). (*C and D*) Growing and serum starvation-induced quiescent HCT116 (*C*) and SW620 (*D*) cells were treated with C212 at the indicated doses for 48 h and subjected to MTS assay. Error bar, SEM (*n* = 3). (E-N) Growing and serum starvation-induced quiescent cells (*E*, HCT116; *J*, SW620) were treated with C212 at the indicated doses for 24 h, followed by Western bolt assay. Protein levels of Hsp90 (*F*, HCT116; *K*, SW620), Hsp70 (*G*, HCT116; *L*, SW620), and Hsp90 client protein p-Erk (*H*, HCT116; *M*, SW620) were normalized to β-actin loading control and plotted against C212 doses. Also shown are p-Erk levels normalized to corresponding Hsp90 levels at tested C212 doses (*I*, HCT116; *N*, SW620). Error bar, SEM (n = 2); * *p* < 0.05, ** *p* < 0.01, *** *p* < 0.001 (over control at 0 μM). **Fig. S5.** The effects of 17-AAG versus C212 on leukemia cells. Growing and serum starvation-induced quiescent (as in Fig. 4 a and b) HL60 (*A*) and K562 (*B*) cells were treated with 17-AAG at the indicated doses for 48 h, followed by MTS assay. Error bar, SEM (n = 3). Growing HL60 (*C-I*) and K562 (*J-P*) cells were treated with C212, 17-AAG, or their combination at the indicated doses for 24 h, followed by Western blot assay. Protein levels of Hsp90 (*D-F*, HL60; *K-M*, K562) and client protein p-Erk (*G-I*, HL60; *N-P*, K562) were normalized to β-actin loading control and plotted against the treatment doses of C212, 17-AAG, or their combination. Error bar, SEM (n = 2); * *p* < 0.05, ** *p* < 0.01, *** *p* < 0.001 (over control at 0 μM).

## Data Availability

All the data generated or analyzed for this project are included either in this article or in the supplementary information files.

## Abbreviations

Hsp90: Heat shock protein 90
MMP: Mitochondrial membrane potential
PARP: Poly (ADP-ribose) polymerase
Cdc2: Cyclin dependent kinase 1
IC50: The concentration required to inhibit cell growth/viability by 50%
