# Supplementary material for: Curcumin derivative C212 inhibits Hsp90 and eliminates both growing and quiescent leukemia cells in deep dormancy
Source: Cell Commun Signal. 2020 Sep 29;18:159. doi: 10.1186/s12964-020-00652-4 (PMC7523331; doi:10.1186/s12964-020-00652-4)
Supplement: Supplementary file 2 — Additional file 1 : Fig. S1. C212 induces G2/M cell accumulation. (A) Chemical structures of curcumin and C212. (B and C) Growing leukemia cells were treated with C212 at the indicated doses for 24 h and subjected to cell-cycle analysis with PI staining of DNA content (B, K562; C, HL60). Error bar, SEM (n = 2); * p < 0.05, ** p < 0.01 (over control at 0 μM). (D) Cells were treated with C212 as in B and C and subjected to Western blot of Cdc2 and cyclin B1 proteins (left, K562; right, HL60). Fig. S2. C212 binds to Hsp90 and induces proteasome-dependent client protein degradation. (A) Molecular docking simulation of the binding of curcumin, C212, and 17-AAG to Hsp90 at its C-terminus, middle (M-)region, and N-terminus. (B) Growing HL60 cells were pretreated with or without C212 (2.4 μM) for 2 h, followed by co-treatment with or without MG132 (1 μM) for 12 h; whole-cell lysate was then subjected to Western blot. Fig. S3 Growing and serum starvation-induced quiescent HL60 (A) and K562 (H) cells were treated with C212 at the indicated doses for 24 h, followed by Western bolt assay. Protein levels of Hsp90 (B, HL60; I, K562), Hsp70 (C, HL60; J, K562), and Hsp90 client protein p-Erk (D, HL60; K, K562) were normalized to β-actin loading control and plotted against C212 doses. Also shown are p-Erk levels normalized to corresponding Hsp90 levels at tested C212 doses (E, HL60; L, K562). Insets in D-L show the enlarged bar graphs corresponding to Quiescence. Error bar, SEM (n = 2); * p < 0.05, ** p < 0.01, *** p < 0.001 (over control at 0 μM). Fig. S4 HCT116 (A) and SW620 (B) cells were serum-starved for 12 and 24 h with EBSS, respectively, to induce quiescence/slow-growth; EdU (10 μM) was added to the medium, and cells were further incubated for 24 h, followed by EdU incorporation assay. Growing: control cells not deprived of serum but otherwise under the same treatment. Error bar, SEM (n = 2). (C and D) Growing and serum starvation-induced quiescent HCT116 (C) and SW620 (D) c [file 12964_2020_652_MOESM2_ESM.pdf]

Figure S1

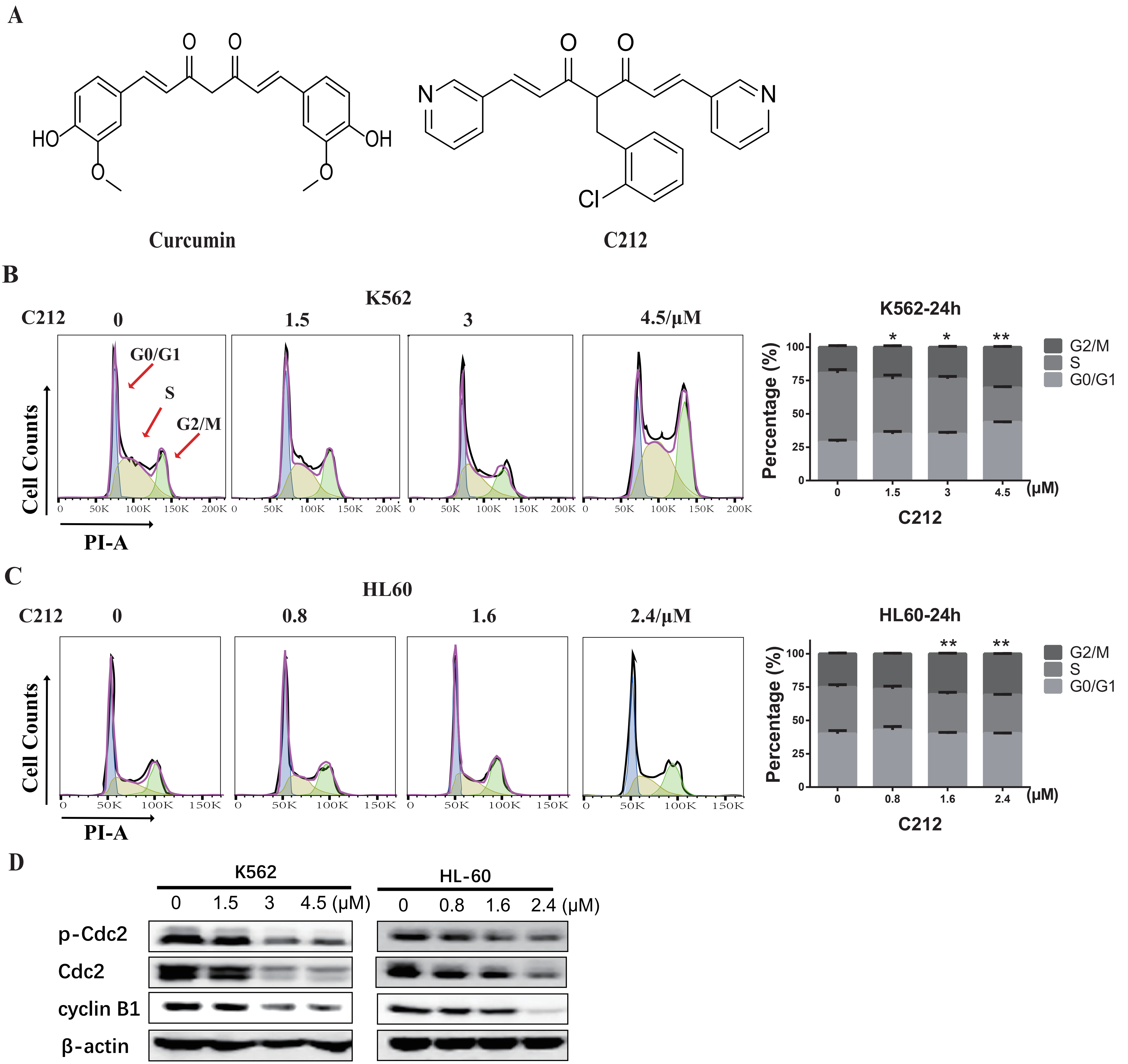

A
O
O
HO
OH
O
O
Curcumin
O
O
N
N
Cl
C212
B
K562
C212
0
1.5
3
4.5/μM
G0/G1
S
G2/M
Cell Counts
PI-A
K562-24h
Percentage (%)
G2/M
S
G0/G1
C212
(μM)
C
HL60
C212
0
0.8
1.6
2.4/μM
Cell Counts
PI-A
HL60-24h
Percentage (%)
G2/M
S
G0/G1
C212
(μM)
D
K562
0
1.5
3
4.5 (μM)
HL-60
0
0.8
1.6
2.4 (μM)
p-Cdc2
Cdc2
cyclin B1
β-actin

Figure S2

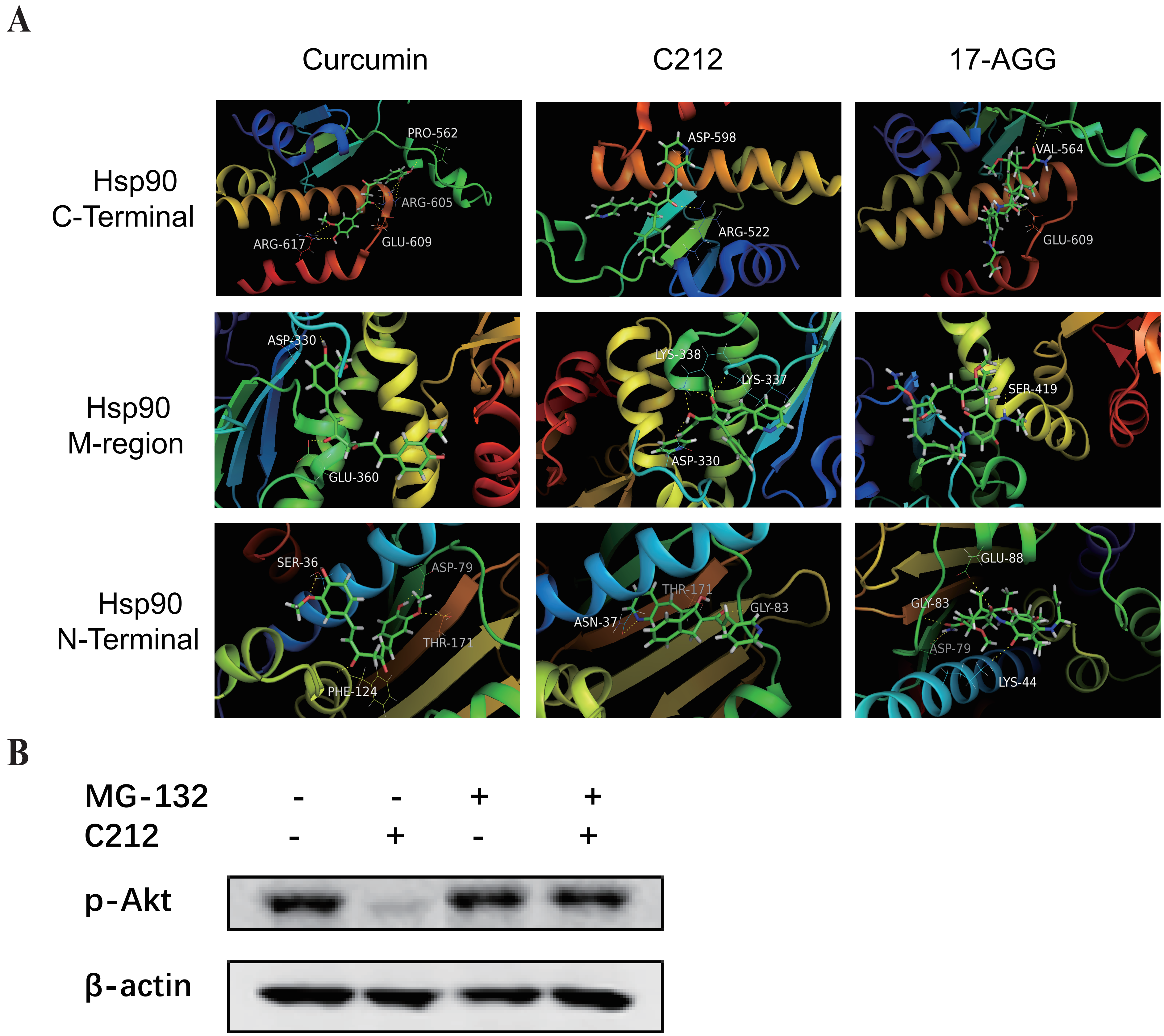
A
Curcumin
C212
17-AGG
Hsp90
C-Terminal
Hsp90
M-region
Hsp90
N-Terminal
PRO-562
ARG-605
ARG-617
GLU-609
ASP-598
ARG-522
VAL-564
GLU-609
ASP-330
GLU-360
LYS-338
LYS-337
ASP-330
SER-419
SER-36
ASP-79
THR-171
PHE-124
THR-171
GLY-83
ASN-37
GLU-88
GLY-83
ASP-79
LYS-44
B
MG-132 - - + +
C212 - + - +
p-Akt
β-actin

Figure S3

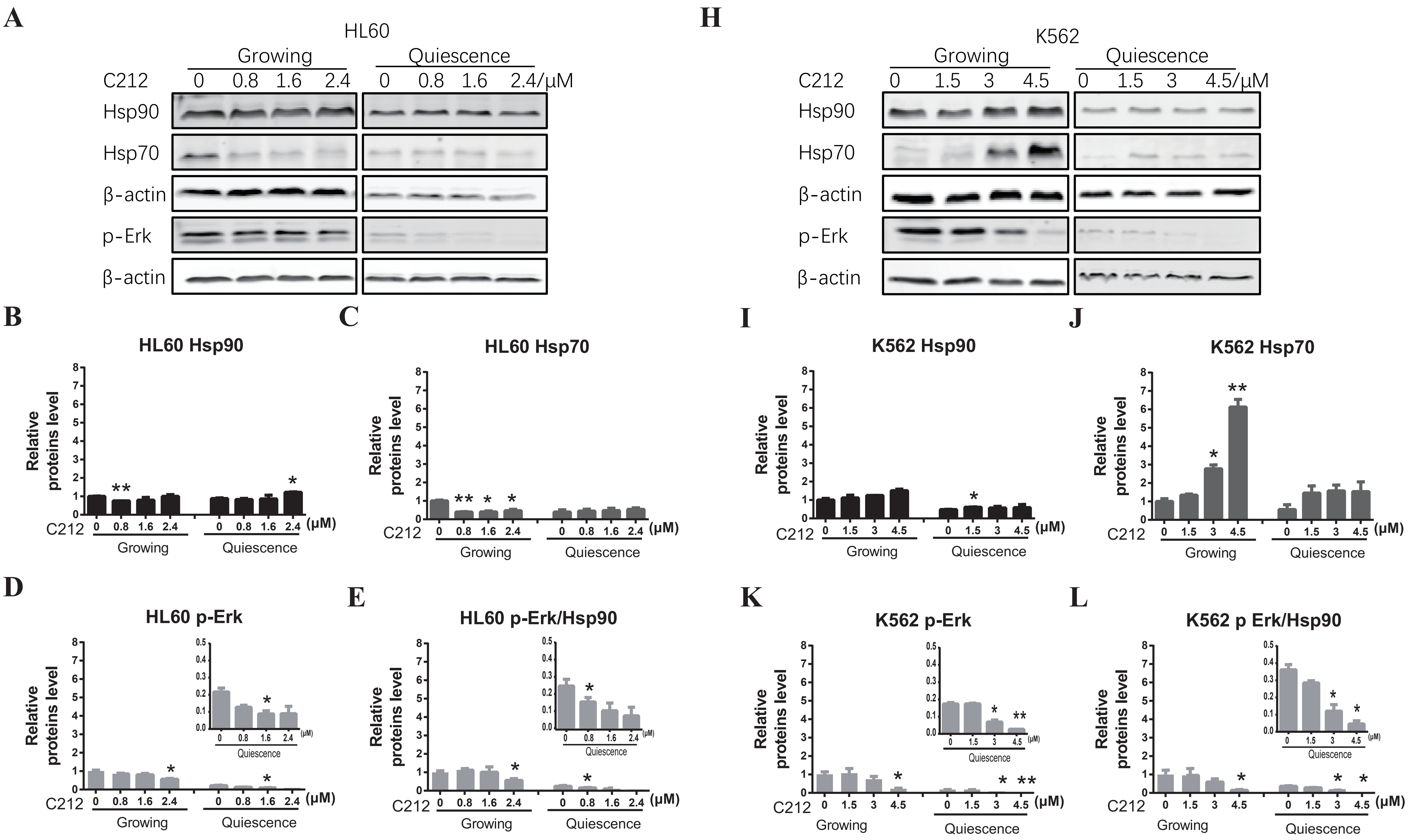

A
HL60
Growing
Quiescence
C212
0 0.8 1.6 2.4
0 0.8 1.6 2.4/μM
Hsp90
Hsp70
β-actin
p-Erk
β-actin
H
K562
Growing
Quiescence
C212
0 1.5 3 4.5
0 1.5 3 4.5/μM
Hsp90
Hsp70
β-actin
p-Erk
β-actin
B
HL60 Hsp90
C
HL60 Hsp70
I
K562 Hsp90
J
K562 Hsp70
D
HL60 p-Erk
E
HL60 p-Erk/Hsp90
K
K562 p-Erk
L
K562 p Erk/Hsp90
Relative proteins level
C212
(μM)
Growing
Quiescence

Figure S4

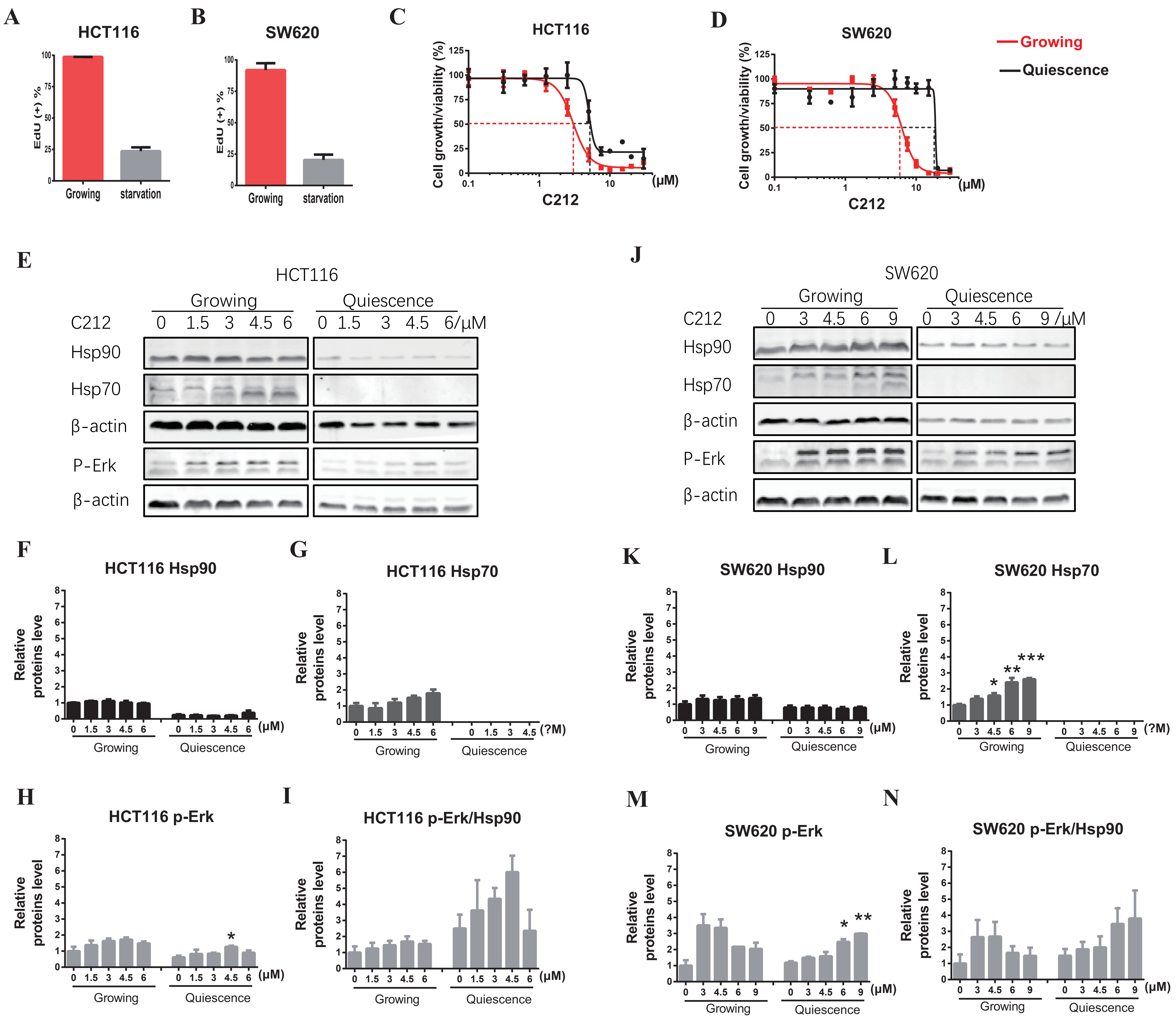

A
HCT116
EdU (+) %
Growing
starvation
B
SW620
C
HCT116
Cell growth/viability (%)
C212
(μM)
D
SW620
Growing
Quiescence
E
HCT116
C212
Hsp90
Hsp70
β-actin
P-Erk
J
SW620
F
HCT116 Hsp90
Relative proteins leve
G
HCT116 Hsp70
Relative proteins level
K
SW620 Hsp90
L
SW620 Hsp70
H
HCT116 p-Erk
I
HCT116 p-Erk/Hsp90
M
SW620 p-Erk
N
SW620 p-Erk/Hsp90

Figure S5

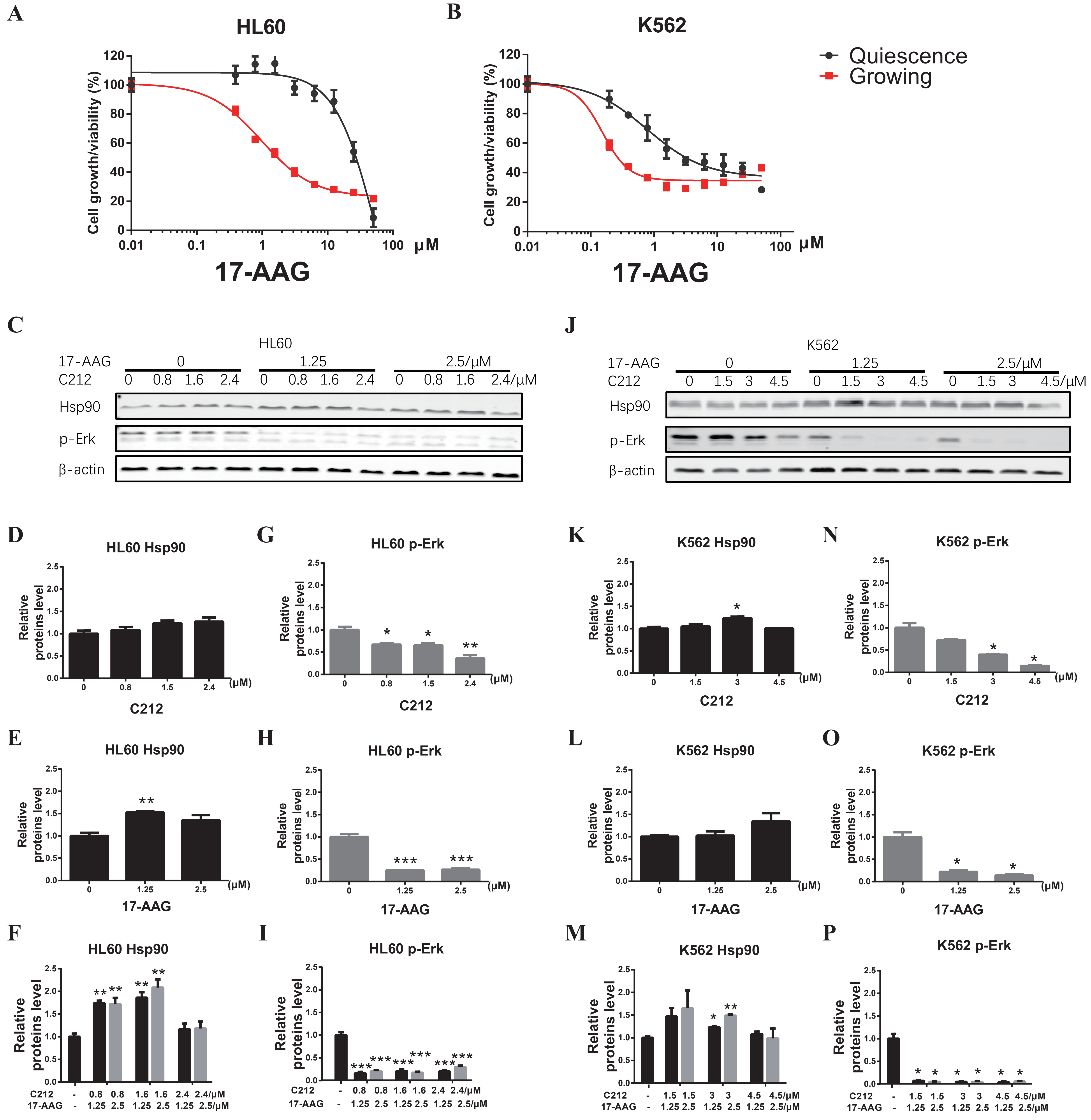

A
HL60
Cell growth/viability (%)
17-AAG
μM
B
K562
Quiescence
Growing
C
HL60
17-AAG
C212
0
1.25
2.5/μM
0 0.8 1.6 2.4 0 0.8 1.6 2.4 0 0.8 1.6 2.4/μM
Hsp90
p-Erk
β-actin
J
K562
0 1.5 3 4.5 0 1.5 3 4.5 0 1.5 3 4.5/μM
D
HL60 Hsp90
Relative proteins level
C212
(μM)
G
HL60 p-Erk
K
K562 Hsp90
N
K562 p-Erk
E
F
H
I
L
M
O
P
